# Perspective: Is Random Monoallelic Expression a Contributor to Phenotypic Variability of Autosomal Dominant Disorders?

**DOI:** 10.3389/fgene.2017.00191

**Published:** 2017-11-29

**Authors:** Baoheng Gui, Jesse Slone, Taosheng Huang

**Affiliations:** ^1^Division of Human Genetics, Cincinnati Children’s Hospital Medical Center, Cincinnati, OH, United States; ^2^Maternal and Child Health Hospital of Guangxi Zhuang Autonomous Region, Nanning, China

**Keywords:** random monoallelic expression, phenotype, expressivity, autosomal dominant disorders, single cell

## Abstract

Several factors have been proposed as contributors to interfamilial and intrafamilial phenotypic variability in autosomal dominant disorders, including allelic variation, modifier genes, environmental factors and complex genetic and environmental interactions. However, regardless of the similarity of genetic background and environmental factors, asymmetric limb or trunk anomalies in a single individual and variable manifestation between monozygotic twins have been observed, indicating other mechanisms possibly involved in expressivity of autosomal dominant diseases. One such example is Holt-Oram syndrome (HOS), which is characterized by congenital cardiac defects and forelimb anomalies, mainly attributed to mutations in the *TBX5* gene. We hypothesize that monoallelic expression of the *TBX5* gene occurs during embryo development, and, in the context of a mutation, random monoallelic expression (RME) can create discrepant functions in a proportion of cells and thus contribute to variable phenotypes. A hybrid mouse model was used to investigate the occurrence of RME with the *Tbx5* gene, and single-cell reverse transcription PCR and restriction digestion were performed for limb bud cells from developing embryos (E11.5) of the hybrid mice. RME of *Tbx5* was observed in approximately two-thirds of limb bud cells. These results indicate that RME of the *Tbx5* gene occurs frequently during embryo development, resulting in a mosaic expression signature (monoallelic, biallelic, or null) that may provide a potential explanation for the widespread phenotypic variability in HOS. This model will further provide novel insights into the variability of autosomal dominant traits and a better understanding of the complex expressivity of disease conditions.

It has been well-recognized that many autosomal dominant Mendelian traits involved in birth defects, adult diseases and other genetic anomalies widely vary in their phenotypic properties such as penetrance, dominance, expressivity, age-of-onset, etc. Although progress has been made in understanding the basis of these features, in the vast majority of cases, it is still not fully revealed why identical genotypes can generate subtly or even profoundly different phenotypes. Several possible factors have been proposed as contributors to interfamilial and intrafamilial phenotypic variability in these genetic disorders, including allelic variation (Zlotogora et al., 1995; Li et al., 2008), modifier genes (Howeler et al., 1989; Riordan and Nadeau, 2017), environmental factors and complex genetic and environmental interactions (Gennari et al., 2010). However, regardless of the similarity of genetic background and environmental effects, asymmetric limb or trunk anomalies in a single individual (Basson et al., 1999; Elliott et al., 2005; Wei et al., 2012) and variable manifestation between monozygotic twins have been observed (Huang, 2002; Huang et al., 2002; Dayer et al., 2007; Mukherjee et al., 2014), indicating other mechanisms are involved in the expressivity of these genetic diseases.

What, then, is the mechanism for this variability? A closer look at the mechanisms of gene expression may reveal some insights. For diploid organisms inheriting two homologous alleles from each parent, the expression of a gene can be carried out in a biallelic (expression of both alleles) or monoallelic (expression of only one allele) manner. In the traditional model, both alleles are expressed simultaneously at similar levels in cells; in reality, however, there are a number of genes that exhibit monoallelic gene expression. A typical case of monoallelic expression is genomic imprinting, in which gene expression occurs from only one allele based on the parental origin of the allele. This phenomenon occurs as a consequence of epigenetic marking of the parental germlines, such as DNA methylation or histone modification, with the result that these genes are expressed exclusively from either the maternal or paternal allele in most somatic cells (Ferguson-Smith et al., 1993; Hu et al., 1998; Carr et al., 2007). Although the evolutionary forces leading to this genetic conflict between parental alleles is still a matter of debate, imprinting is known to be a particularly critical factor in mammalian development, with experiments dating back to the 1980s clearly showing that both maternal and paternal genetic contributions are required for the completion of embryogenesis (Barton et al., 1984; McGrath and Solter, 1984). Incorrect imprinting of specific genomic loci has also been demonstrated to be correlated with disease conditions, such as Angelman and Prader-Willi syndromes (Buiting et al., 1995).

Another distinct class of monoallelic expression is random monoallelic expression (RME), defined as the stochastically determined, selective expression of a single allele. Random X-chromosome inactivation (XCI) is a well-documented subset of RME (Lyon, 1961, 1962, 1989; Smith, 1985; Wu et al., 2014; Chen et al., 2016). In female cells, one copy of the X-chromosome is randomly silenced; as a result, alleles located on the remaining active chromosome are monoallelically expressed. The ultimate outcome of this process is that the X-chromosome gene “dosage” between male and female cells becomes roughly equal, preventing the severe developmental and metabolic problems that would result from an entire chromosome becoming transcriptionally unbalanced between the sexes (Smith, 1985; Lyon, 1989). In addition to XCI, RME occurring on autosomes has been studied, especially in large gene families with functions related to the nervous and immune systems, such as the olfactory receptor gene family (Chess et al., 1994; Rodriguez, 2013), protocadherins (Esumi et al., 2005), and immunoglobulins (Pernis et al., 1965). Intriguingly, increasing numbers of studies have also revealed that autosomal RME can occur in individual genes (Bix and Locksley, 1998; Hollander et al., 1998; Gimelbrant et al., 2005; Calado et al., 2006; Takizawa et al., 2008; Thomas et al., 2011; Aseem et al., 2013) outside of these large gene families. These scattered genes are involved in a wide range of cellular functions, and monoallelically expressed in distinct types of cells.

Since monoallelically expressed genes are prevalent genome-wide and involved in a wide range of functions (Gimelbrant et al., 2007; Deng et al., 2014), RME might be closely related to gene expression regulation and tuning, cell differentiation, and/or embryo development. Any of these would place RME in a position to significantly influence phenotypic variability and disease status. Physiologically, RME has the potential to generate different transcriptional signatures and profiling, resulting in a remarkable level of cellular diversity. A typical and well-studied case is the olfactory receptor gene family, which consists of approximately 1400 functional genes in mice and 350 in humans, scattered across 40 or more genomic clusters (Young et al., 2002; Clowney et al., 2011). It has been demonstrated that these receptor genes are expressed in a monogenic (Buck and Axel, 1991) and monoallelic (Chess et al., 1994) manner in the main olfactory epithelium, with the latter likely helping to reinforce the former. This strict expression of a single allele, from a single gene, out of almost 350 possible loci is quite remarkable, and enables each individual neuron to possess a highly specific and narrowly tuned range of odor recognition. Also, given that correct axon guidance of olfactory neurons is dictated by the odorant receptors they express (Wang et al., 1998), this monoallelic expression pattern is critical to the existence of a well-organized and harmonious olfactory network. In the realm of more serious genetic disorders, RME could provide an intriguing explanation for the unexplained variability in pathology and clinical symptoms for autosomal dominant genetic diseases. Theoretically, RME could cause phenotypic variability with regards to penetrance and expressivity, either through the dosage difference between expressing one rather than two homologous alleles, or by stochastically initiating expression of a non-functioning allele when the genotype is heterozygous (Reinius and Sandberg, 2015). However, in order to entertain the possibility that RME is behind these phenotypic variabilities, what is first required is an autosomal dominant disease model amenable to a cell-by-cell investigation of allelic expression.

Among those autosomal dominant disorders with variable expressivity is Holt-Oram syndrome (HOS). This disorder is characterized by congenital cardiac defects and forelimb anomalies, mainly attributed to mutations of the *TBX5* gene, a member of the T-box family of transcription factor genes (Basson et al., 1994, 1997). The clinical manifestations of HOS vary widely (Basson et al., 1994, 1999; Newbury-Ecob et al., 1996; Sletten and Pierpont, 1996; Brassington et al., 2003), and these interfamilial and intrafamilial phenotypic variations have been explained by the hypothesis that modifier genes or other aspects of the genetic background may play an important role. Previous molecular studies tried to establish initial genotype–phenotype correlations and have indicated that mutations predicted to create truncated TBX5 can produce substantial abnormalities in both the limbs and heart. In contrast, missense mutations of *TBX5* could result in two distinct categories of phenotypes, depending on their location in the T box: either significant cardiac malformations (but only minor skeletal abnormalities), or more extensive upper limb malformations (but less significant cardiac abnormalities) (Basson et al., 1999). However, further analysis of the expressivity of HOS in a larger cohort with more independent cases has suggested that neither the type of mutation in *TBX5* nor the location of a mutation in the T box could accurately predict the phenotypic variability in individuals with the condition (Brassington et al., 2003). Moreover, given that many of these cases involve asymmetric limb anomalies within a single individual (Basson et al., 1999) and variable phenotypes have been observed even in monozygotic twins (Huang, 2002; Huang et al., 2002), it is clear that genetic background and environmental effects alone cannot reasonably explain the discordant features in individuals with HOS. Thus, the molecular mechanisms that lead to wide phenotypic variability in HOS remain poorly understood. As stated above, however, we believe that the proposed model of RME-mediated disease may explain this variability, and have utilized the *TBX5* gene as a means to test this model. We hypothesize that monoallelic expression of the *TBX5* gene occurs during embryo development, contributing to the fine-tuning of developmental regulatory pathways. In the context of a mutation, this would suggest that RME can create discrepant functions in a proportion of cells. This, we propose, contributes to discordant features and variable phenotypes, such as asymmetric malformations.

A hybrid mouse model was used to investigate the occurrence of RME in the mouse ortholog of the *Tbx5* gene. A single nucleotide polymorphism (SNP) site has been identified in the 3′ untranslated region of the mouse *Tbx5* gene to distinguish different parental alleles. Specifically, C3H/HeJ and BALB/cJ mice carry a homozygous T and C nucleotide at c.2583 (NM_011537.3) of the gene, respectively. Embryos of F1 heterozygous mice were obtained from the following mouse cross: C3H/HeJ × BALB/cJ. Single-cell reverse transcription (RT)-PCR and restriction digestion were performed for limb bud cells from the developing F1 embryos at the E11.5 stage, during which the *Tbx5* interacts with other factors, such as *Sall4*, to initiate the complex regulation of limb patterning and morphogenesis in mouse embryos (Koshiba-Takeuchi et al., 2006). The limb bud cells were collected from both the left and the right forelimbs and digested with collagenase and trypsin. After the cells were dispersed into single cell suspension by pipetting, a fluorescence-activated cell sorter (BD FACSAria II, BD Biosciences, San Jose, CA, United States) was used to sort the cells according to the manufacturer’s instructions, and single cells were placed into 96-well plates. The single cells were lysed and treated with DNase (Ambion Turbo DNAfree, Austin, TX, United States). An RT-PCR was performed with a primer pair that spans the last two exons of the *Tbx5* gene in order to exclude contamination from residual genomic DNA using a single-step RT-PCR kit (OneStep RT-PCR Kit, QIAGEN, Hilden, Germany), and the products were then subjected to a nested PCR to amplify the target fragment in the 3′ untranslated region of *Tbx5*. The final products underwent restriction endonuclease digestion using *Bsa*OI (recognition sequence: CGRYCG), which can distinguish the heterozygous genotype of T/C. For the paternally expressed allele (BALB/cJ with the sequence: CGACCG), the PCR products were specifically cut into two smaller fragments, 153 and 108 bp, while PCR products with a full length of 261 bp were observed for the maternally expressed allele (C3H/HeJ with the sequence: TGACCG). In total, 38 single limb cells were gathered, and readable results were obtained for 30 samples, with the other eight showing no readable signals. The unreadable signals were likely due to either low transcriptional levels of the *Tbx5* gene in those particular cells, or failure of single cell lysis or nested RT-PCR. Among the readable signals, 60% (18 out of 30) showed monoallelic expression model of *Tbx5*, in which 23.3 and 36.7% of alleles (7 and 11 out of 30, respectively) were paternally and maternally expressed, respectively (**Figure 1**).

**FIGURE 1:**
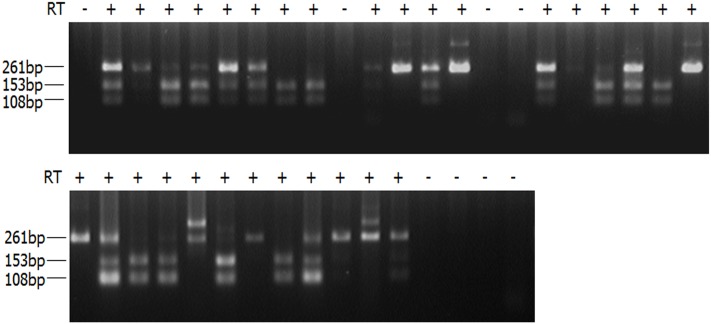
RT-PCR products of single limb bud cells digested with *Bsa*OI. Single-cell RT-PCR products of limb bud cells were digested with *Bsa*OI (recognition sequence: CGRYCG). For paternally expressed allele (BALB/cJ with the sequence: CGACCG), the PCR products were specifically cut into two smaller fragments, 153 and 108 bp, while the PCR products with a full length of 261 bp were observed for the maternally expressed allele (C3H/HeJ with the sequence: TGACCG). PCR products displaying all three bands indicate biallelic expression in those particular cells. PCR products showing no readable signals are likely due to either low transcriptional level of the *Tbx5* gene that fall below the detection threshold in single cells, or failure of single-cell lysis or nested RT-PCR.

Through the hybrid mouse model, this study found that *Tbx5* was monoallelically expressed in about two-thirds of limb bud cells. The monoallelic expression is random and independent of parental origin. Since the E11.5 mouse limb buds have differentiated into several cell types with early hints of apical ectodermal ridge, humerus, myogenic cells, nerve fascicles, etc. (Martin, 1990), it is certainly possible that the observed monoallelic expression pattern could be attributable to tissue-specific expression. Alternatively, both the random monoallelic and the tissue-specific models could be true, depending on the cell type in question (e.g., skin cells may only express one allele, while muscle cells express both alleles in a random monoallelic fashion). Regardless, our results indicate a generally monoallelic expression pattern and a mosaic expression signature (monoallelic, biallelic, or null) of Tbx5 in mouse developing limb bud cells within the critical development window. As a control, we also examined cardiomyocytes with a similar approach. We found that all of the cardiomyocytes expressed both alleles due to the natural presence of multiple nuclei in fetal cardiomyocytes. These findings may provide a reasonable explanation for the discordant limb malformations in monozygotic twins with the same *TBX5* genotype and asymmetric limb anomalies in individuals with HOS.

Random monoallelic expression could cause widespread phenotypic variability in autosomal dominant disorders by stochastically initiating expression for one of the two functionally discrepant parental alleles (Reinius and Sandberg, 2015). This has been well-discussed in X-chromosome linked diseases, in which males are hemizygous and show more severe disease manifestation than females, since females are cellular mosaics for X-linked gene profiles. This does not mean, of course, that females escape the consequences of these mutations unscathed, as a subset of their tissues can become affected depending on whether the X-chromosome containing the mutant allele or the one containing the wide-type allele is chosen for random silencing during early embryonic development. For instance, mutation of the X-linked gene *MECP2*, encoding the “methyl-CpG binding protein 2,” is lethal for males at an early age, while females with mutations can survive, but often develop Rett syndrome, a severe progressive neurological disorder (Amir et al., 1999). In fact, even in monozygotic twins, the implications can be quite different. In a previous study of a pair of monozygotic twins, one of the twins was affected by Rett syndrome, while the other one showed no observable manifestations (Migeon et al., 1995). The *MECP2* gene is dosage-sensitive, and thus plays a critical role in a disease context. For instance, duplications of *MECP2* also cause severe mental retardation in males (Van Esch et al., 2005), and mild to severe mental retardation in females (Grasshoff et al., 2011). Importantly, it has been indicated that several autosomal RME genes, whose dosage are essential for gene function, are associated with various disorders. APP (amyloid beta precursor protein) and SNCA (alpha-synuclein), which are implicated, respectively, in Alzheimer’s (Rovelet-Lecrux et al., 2006) and Parkinson’s diseases (Singleton et al., 2003), are a case in point (Gimelbrant et al., 2007; Eckersley-Maslin et al., 2014; Gendrel et al., 2014). The high levels of expression of these genes are known to be detrimental, and disturbing their RME regulation likely enhances the dosage of these genes and potentially triggers diseases.

Few studies have explored the possible link between RME and the penetrance or expressivity of autosomal dominant inherited diseases. In previous studies, the eyes absent homolog 1 (*Eya1*) gene and sine oculis homeobox homolog 1 *(Six1)* gene were indicated to undergo stable RME in mouse neural progenitor cells, and *Eya1* was found to be monoallelically expressed *in vivo* during mouse cochlear development (Gendrel et al., 2014). Forming a bipartite complex, the EYA1 and SIX1 proteins function as transcriptional activators, and play essential roles in organogenesis during embryo development. Loss of function of the EYA1-SIX1 complex causes Branchiootorenal syndrome (Ruf et al., 2004; Zou et al., 2008), an autosomal dominant disorder characterized by craniofacial abnormalities, hearing loss, and kidney deficiency, but with widely varying conditions and phenotypes, even among individuals from the same family. *Eya1* gene dosage critically affects the development of sensory epithelia in the mammalian inner ear, and different threshold levels of *Eya1* are required in different regions of the inner ear (Zou et al., 2008). In mice, *Eya1* homozygous mutant individuals lack ears and kidneys, while heterozygous mutants exhibit a conductive hearing loss and renal abnormalities, similar symptoms to the Branchiootorenal syndrome (Xu et al., 1999). These findings suggest that RME of dosage-sensitive genes, such as *EYA1* and *SIX1*, could potentially play a vital role in phenotypic variability, either through haploinsufficiency or a total absence of functional protein in a critical portion of cells attributable to random inactivation of the wide-type allele. Given that the random and dynamic expression of RME genes potentially generates a mosaic expression signature, the ratio between cells expressing mutant alleles versus wild-type alleles in various tissues or organs, together with the timing of these expression patterns at specific developmental stages, are likely to be significant factors in autosomal dominant diseases with regards to their penetrance and expressivity (**Figure 2**).

**FIGURE 2:**
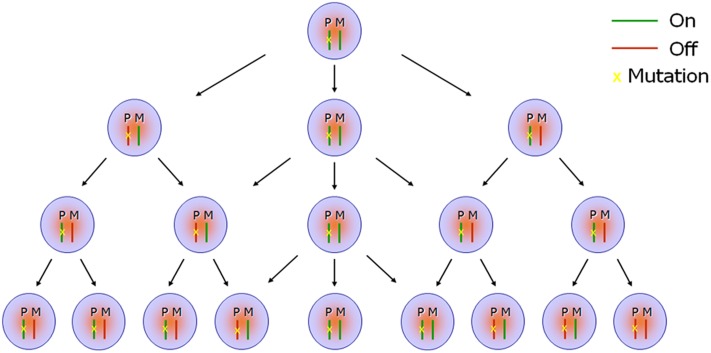
Scheme of monoallelic expression in developing organs. Random monoallelic expression (RME) occurs in maternal or paternal alleles (i.e., turned “On” or turned “Off”) stochastically in the developing embryos, which results in a mosaic expression signature (monoallelic, biallelic, or null) in a specific tissue or across different tissues. When one of the parental alleles is mutant—for example, the paternal allele as indicated in the scheme (P: paternal; M: maternal)—the mosaic expression profile further generates co-existence of discrepant protein composition or function in a proportion of cells, attributable to random inactivation of the wild-type maternal allele. For instance, cells showing haploinsufficiency for a particular gene will co-exist alongside cells completely lacking functional protein when the gene mutation is loss-of-function.

With the development of high-throughput screening technologies, such as SNP arrays (Gimelbrant et al., 2007; Jeffries et al., 2012; Zwemer et al., 2012) and next-generation RNA-sequencing methodologies (Nag et al., 2013; Xue et al., 2013; Deng et al., 2014; Eckersley-Maslin et al., 2014; Gendrel et al., 2014; Marinov et al., 2014; Borel et al., 2015), RME has been studied and assessed at a transcriptome-wide level. Gimelbrant et al. (2007) performed the first genome-wide analysis of RME in clonal populations of human lymphoblast cell lines by SNP arrays and found that 9.4% of genes (371 out of 3939 assessable genes) were monoallelically expressed based on at least one informative SNP. A similar ratio of RME genes (15.6%, or 212 out of 1385 assessed genes) was found in mouse B lymphoblasts (Zwemer et al., 2012). Compared with lymphoblasts, relatively fewer genes (approximately 2 to 3%) were found to be expressed in a monoallelic pattern in neural stem and progenitor cells (Jeffries et al., 2012; Eckersley-Maslin et al., 2014; Gendrel et al., 2014). In fact, among the assessed autosomal genes in human or mouse, while as many as 5 to 10% of the genes in lymphoblasts (Gimelbrant et al., 2007; Zwemer et al., 2012) were mitotically stable across different clones and generations, a relatively low level were found to meet this criteria in fibroblasts (Zwemer et al., 2012), neural stem cells (Jeffries et al., 2012) and progenitor cells (Eckersley-Maslin et al., 2014; Gendrel et al., 2014), and embryonic stem cells (Eckersley-Maslin et al., 2014). However, RME could also be a dynamic process, in which the monoallelic expression is temporary and not conserved during mitosis, likely resulting from unsynchronized or discrete transcriptional bursting of the two alleles (Xue et al., 2013; Deng et al., 2014). Recent single-cell RNA-sequencing studies have discovered widespread dynamic monoallelic expression in human and mouse (Xue et al., 2013; Deng et al., 2014; Marinov et al., 2014; Borel et al., 2015). These investigations identified abundant RME of autosomal genes in the mouse *in vivo* preimplantation embryos (12 to 24% of genes), *in vivo* hepatocytes (∼30% of genes) and *in vitro* fibroblasts (∼24% of genes) (Deng et al., 2014). Dynamic RME has also been observed in human lymphoblastoid cells (Marinov et al., 2014) and primary fibroblasts (Borel et al., 2015). The application of high-throughput screening technologies in further studies of RME genes will facilitate a better understanding of gene transcriptional regulation and its relationship with the expressivity and severity of genetic disorders.

In conclusion, we have observed RME of the *Tbx5* gene during mouse embryo development, which may provide a potentially reasonable explanation for the widespread phenotypic variability in HOS. Based on this result and the review of the relevant literature described above, we suggest that the possibility of RME should be investigated in those situations where autosomal dominant disorders display intra- and interindividual variability. This model will further provide novel insights into the variability of autosomal dominant traits, and a better understanding of the expressivity of disease conditions.

## Ethics Statement

Thisstudy wascarried out in accordance with the recommendations of the Cincinnati Children’s Institutional Animal Care and Use Committee. The protocol was approved by the Cincinnati Children’s Institutional Animal Care and Use Committee.

## Author Contributions

TH conceived the idea for this study. BG and TH analyzed and interpreted the results, BG, TH, and JS wrote the manuscript and revised it, and approved the final version to be published. The project was designed and supervised by TH.

## Conflict of Interest Statement

The authors declare that the research was conducted in the absence of any commercial or financial relationships that could be construed as a potential conflict of interest.
